# The Impact of Regional Maximum Tolerated Interlesion Distance on the Long-Term Ablation Outcomes in Ablation Index Guided Pulmonary Vein Isolation for Atrial Fibrillation

**DOI:** 10.3390/jcm12155056

**Published:** 2023-08-01

**Authors:** Radoslaw M. Kiedrowicz, Maciej Wielusinski, Wojciech Krasnik, Olga Jankowska, Szymon Zakrzewski, Lukasz Duda, Małgorzata Peregud-Pogorzelska, Aleksandra Kladna, Jaroslaw Kazmierczak

**Affiliations:** 1 Cardiology Department, Pomeranian Medical University, Powstancow Wlkp. 72, 70-111 Szczecin, Polandjankowskaolga.jo@gmail.com (O.J.);; 2 Department of History of Medicine and Medical Ethics, Pomeranian Medical University, Rybacka 1, 70-204 Szczecin, Poland

**Keywords:** atrial fibrillation, pulmonary vein isolation, interlesion distance

## Abstract

Background: An adequate interlesion distance (ILD) applied during point-by-point pulmonary vein (PV) isolation for atrial fibrillation (AF) has never been established. We hypothesized that maximum tolerated ILD may differ between PV regions and may influence long-term ablation outcomes. Methods: A total of 260 AF patients underwent PV isolation with 3D electroanatomical platform. Postablation, ILD values were classified into 5 groups (6–5.5 mm, 5.5–5.0 mm, 5.0–4.5 mm, 4.5–4.0 mm and <4.0 mm); the number of tags in each group was calculated and correlated with postablation AF recurrence (AFR). All measurements were performed globally for the entire encirclement around each individual PV and regionally for designated PV anatomic segments. Results: Single-procedure freedom from AF was 81% for paroxysmal and 66% for persistent AF at 12 months. Global analysis showed that AFR was not related to median ILD nor the number of lesions within each ILD tag group for any PV. Segmental analysis showed that *AFR* was not related to median ILD. However, the presence of tags from the 5.5–6.0 mm ILD group located on the posterior aspect of right upper PV (RUPV) correlated with AFR. This was confirmed in a multivariable logistic regression model. Conclusions: Maximum tolerated ILD of 6.0 mm translated into well-accepted ablation results. However, the study suggests that it may be inadequate at the posterior aspect of RUPV.

## 1. Introduction

Late pulmonary vein (PV) reconnection following point-by-point pulmonary vein isolation (PVI) is considered as the major determinant of atrial fibrillation (AF) ablation failure. Lack of PVI durability is usually explained by the incomplete transmurality or discontiguity of RF lesions within deployed PV encirclement [1]. The intraprocedural measurement of interlesion distance (ILD) and prediction of the lesion extent, calculated using ablation duration, catheter contact and delivered power, combined into a weighted formula, currently provides the best strategy to predict durable PVI. Well-accepted data promote an ILD ≤ 6 mm throughout each PV encirclement [2,3]. Nevertheless, adequate ILD has never been established; a growing body of evidence suggests that closer ILD should be targeted [4,5,6,7,8,9]. It was hypothesized that maximum tolerated ILD may differ between PV regions, due to variations in lesion characteristics being dependent on anatomic location [8]. Based on this hypothesis, ILD ≤ 6 mm may be sufficient in some anatomical PV segments. However, closer ILD may be necessary at others. Therefore, we aimed to investigate whether regional ILD may influence long-term ablation outcomes.

## 2. Materials and Methods

### 2.1. Study Population

The cohort study included 260 consecutive AF patients, 130 with paroxysmal (PAF) and 130 with persistent AF (PsAF), who underwent first-time RF point-by-point PVI at our center. To minimize the probability of presenting extra PV triggers, only patients without low voltage left atrial (LA) substrate were included. The clinical characteristics of the study population are presented in Table 1. All antiarrhythmic drugs were discontinued for at least five half-lives before ablation. The study protocol was approved by a local institutional review board and all patients provided written informed consent.

### 2.2. Voltage Mapping Protocol

The mapping protocol for detection of low-voltage areas is described in detail elsewhere [10]. Briefly, this was performed during coronary sinus (CS) pacing with a Pentaray catheter (Biosense-Webster [BW], Irvine, CA, USA), acquired with a CONFIDENSE™ module (BW) using CARTO^®^3 electroanatomical platform (BW). EGM amplitude ≥ 0.5 mV was defined as normal and <0.5 mV as diseased tissue. All points presenting low voltage were visually inspected and those incorrectly annotated or presenting far-field signals were deleted from the map. Patients presenting AF were cardioverted into sinus rhythm. Only patients who were able to maintain sinus rhythm underwent high density–high resolution LA bipolar voltage mapping.

### 2.3. Ablation Index and Interlesion Distance Guided PVI

An ablation procedure was performed under conscious sedation. Following creation of LA respiration-gated shell and voltage mapping with the Pentaray catheter, point-by-point ipsilateral circumferential PVI was performed with a 3.5 mm-tip Thermocool SmartTouch SF catheter (BW). A non-steerable sheath (Swartz SL0, Abbott, Plymouth, MN, USA) was used for both mapping and ablation. RF applications were deployed 1 cm away from the PV ostium. Each PV encirclement was complemented with intravenous carina ablation where applicable. The rationale for this strategy was based on an observation that the non-isolation of PV carina following a successful encircling ipsilateral PVI is relatively common and often not detected via the standard mapping approach, and can translate into AF recurrence [11]. Furthermore, reconnection anywhere along ablation encirclement allows veno-atrial conduction from both PVs. The separation of ipsilateral PVs may limit this phenomenon to a single PV.

Six millimeter-diameter ablation tags were automatically generated by the system software (VISITAG, version 6, BW) when the catheter stability was maintained for 3.0 s within a 2.5 mm range, with the minimum contact force of 3 g for 25% of application duration and respiratory adjusted. RF applications were delivered in a power-controlled mode with 40 W until achieving 450 for the ablation index (AI) algorithm value (BW) on the posterior wall and 550 elsewhere, along with the obligatory impedance drop >3 Ω [12]. In order to keep lesion contiguity, we aimed for a real-time ILD of ≤6 mm for each lesion pair. In the case of catheter dislocation or failure to achieve AI, impedance drop or ILD targeted values, additional RF applications were applied. The endpoint of PVI was to document an entrance block verified with the Pentaray catheter, along with the lack of capture distal from the ablation line, inside each individual PV, verified with the ablation catheter presenting contact ≥6 g. Each PV was screened for dissociated potentials following PVI throughout a 1 min downtime. In the case of not achieving first-pass isolation (FPI) or the detection of PV reconnection during a 20 min waiting time, the site of gap or reconnection was allocated based on the earliest Pentaray signal and/or LA–PV junction presenting amplitude >0.2 mV on the bipolar voltage mapping or pacing along the ablation line. Touch-up applications were continued until PVI was resistant to subsequent waiting.

### 2.4. Postprocedural Assessment of Interlesion Distance

**Figure 1:**
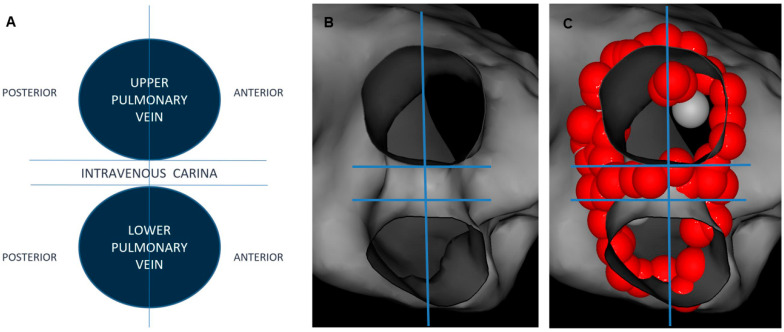
Upper and lower ipsilateral pulmonary veins subdivided (blue lines) into 3 anatomic segments: anterior, posterior and intravenous carina. (**A**) schematic diagram, (**B**) electroanatomical shell without ablation tags (**C**), electroanatomical shell with ablation tags.

**Figure 2:**
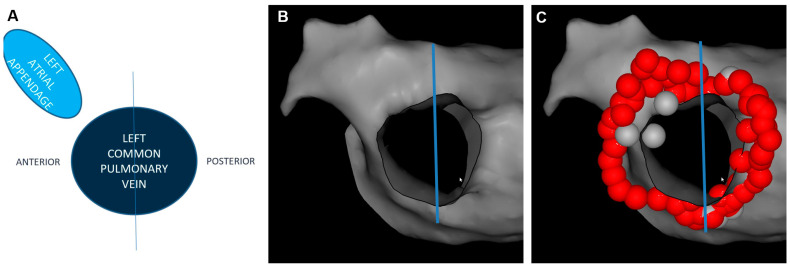
Common left pulmonary vein subdivided (blue line) into 2 anatomic segments: anterior and posterior, due to the lack of intravenous carina. (**A**) schematic diagram, (**B**) electroanatomical shell without ablation tags (**C**), electroanatomical shell with ablation tags.

**Figure 3:**
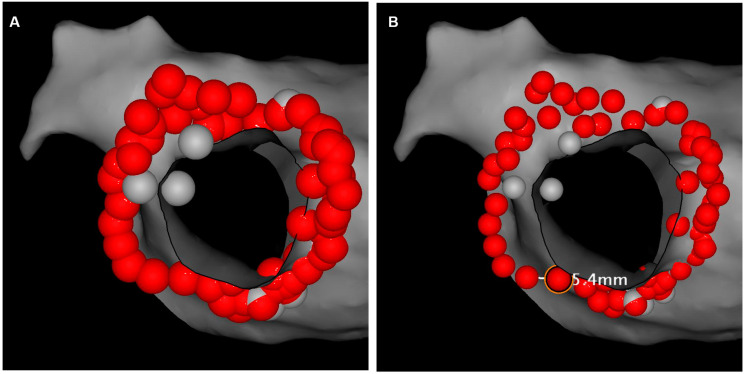
Postprocedural assessment of interlesion distance (ILD) with a custom CARTO^®^3 system software. (**A**) Six millimeter-diameter tags where automatically generated during ablation with a maximum tolerated ILD of 6 mm for each lesion pair. Therefore, all tags were contiguous or overlapping each other. (**B**) RF lesions were downscaled into 4 mm-diameter tags, creating visual gaps ≤ 2 mm between ablation points. ILD was determined by measuring a center-to-center distance between two neighboring ablation tags.

Each individual PV was assessed separately and subdivided into 3 anatomic segments: anterior, posterior and intravenous carina, where applicable (Figure 1 and Figure 2). ILD was determined by measuring a center-to-center distance between two neighboring ablation tags with custom CARTO^®^3 version 6 system software. All tags were contiguous or overlapping with each other; therefore, maximal ILD between 2 neighboring lesions was 6 mm. For better visualization, RF lesions were downscaled offline into 4 mm diameter tags. This manoeuver created some visual gaps ≤2 mm between ablation points (Figure 3). First, the perimeter of deployed encirclement, the total number of 6 mm RF tags and a median ILD value were calculated. ILDs were classified into 5 groups with 0.5 mm steps (6–5.5 mm, 5.5–5.0 mm, 5.0–4.5 mm, 4.5–4.0 mm and <4.0 mm) and the number of RF tags in each group was calculated. All measurements were performed globally for the entire PV and then regionally for designated anatomic segments.

### 2.5. Follow-Up Strategy

All patients were screened over a 1-year period. Outpatient visits were scheduled at 3, 6, 9 and 12 months following ablation. At each visit, a detailed medical history was taken, with emphasis on registered AF episodes or AF suggestive symptoms, along with 24 h Holter monitoring. Freedom from AF was defined as the absence of AF or other sustained (>30 s) atrial tachyarrhythmia after the procedure beyond a 3-month blanking period. No antiarrhythmic drugs were allowed throughout the study.

### 2.6. Statistics

All continuous variables are expressed as median and interquartile range, as they were not normally distributed. The categorical variables are presented as values and percentages. Comparisons between groups were performed with either the Mann–Whitney U-test, the Wilcoxon test or the chi^2^ test, where appropriate. A correlation between variables was assessed using the Spearman rank test. Univariate and multivariate logistic regression analyses were used to determine factors that were associated with a postablation AF recurrence (AFR). Only variables with a *p*-value <0.05 in univariate analysis were included for further evaluation in a multivariate model, using a stepwise forward regression. The receiver operating characteristic analysis was used to determine the optimal cut-off value for predicting AFR. Statistical significance was accepted at *p*-value *<* 0.05. The analysis was performed using Statistica software version 13.3 (StatSoft).

## 3. Results

All PVs were successfully isolated. Single-procedure freedom from AF was 81% and 66% among PAF and PsAF patients, respectively (*p* < 0.001). Significant clinical and intraprocedural differences between PAF and PsAF cohorts were noted; especially, larger LA size in the PsAF subgroup transformed into longer ablation encirclement and, finally, more RF tags around each PV (Table 1). Therefore, a detailed analysis was made separately for PAF and PsAF subgroups. However, no differences in total median ILD [4.4 mm (4.3–4.5) in PAF and 4.4 mm (4.2–4.5) in PsAF], median ILD applied around individual PVs or the achievement of FPI were noted between AF cohorts. Moreover, within both the PAF and PsAF subgroups, ILD was significantly lower and the number of 6 mm RF tags significantly higher when ablation was applied around LCPV, compared to the other PVs. This transformed into the highest level of FPI among all PVs. Furthermore, the perimeter of encirclement did not differ between each PV (Table 1).

### 3.1. Paroxysmal AF

Global analysis, assessing the entire encirclement around each individual PV, showed that AFR was not related to median ILD nor the number of lesions within each ILD tag group for any PV.

Segmental analysis for each designated anatomical PV region showed that *AFR* was not related to median ILD. However, the presence of tags from the 5.5–6.0 mm ILD group located on the posterior aspect of RUPV correlated with AFR [20/25 (80%) AFR+ vs. 32/105 (30%) AFR−; *p* < 0.001]. The presence of a single 5.5–6.0 mm ILD tag in this area predicted AFR with 83% sensitivity and 78% specificity (Figure S1—Supplementary Material). This was confirmed in a multivariable logistic regression model where only 5.5–6.0 mm ILD tags remained independently predictive of AFR (OR = 1.73, 95% CI = 1.17–2.36, *p* < 0.001).

### 3.2. Persistent AF

Global analysis showed that AFR was not related to median ILD and the number of lesions within each ILD tag group when assessed for the left upper, left lower and right lower PV. The presence of 5.5–6.0 mm ILD tags around right upper PV (RUPV) significantly correlated with AFR (*p* = 0.01) but not median ILD. An ROC curve analysis showed that the presence of a single 5.5–6.0 mm tag predicted AFR with 71% sensitivity and 55% specificity. However, a multivariable logistic regression analysis revealed that no covariates were independently predictive of AFR.

Segmental analysis showed that only 5.5–6.0 mm tags located on the posterior aspect of RUPV correlated with AFR [38/44 (86%) AFR+ vs. 17/86 (20%) AFR−; *p* < 0.001]. The presence of a single tag in this area predicted AFR with 90% sensitivity and 80% specificity (Figure S2—Supplementary Material). This was confirmed in a multivariable logistic regression model where only 5.5–6.0 mm ILD tags remained independently predictive of AFR (OR = 1.84, 95% CI = 1.19–2.87, *p* < 0.001). However, AFR was not related to median ILD.

The perimeter of the deployed encirclement, number of 6 mm RF tags and presence of dissociated potentials following PVI did not correlate with AFR in the global and segmental analysis among both PAF and PsAF subgroups. Median ILD values and median number of lesions within each tag group among the AFR and non-AFR group are shown in Table 2, Table 3, Table 4 and Table 5.

## 4. Discussion

Ablation lesion contiguity is one of the critical determinants of procedural success [1]. Based on the findings of the CLOSE protocol, a maximally tolerable ILD to achieve PVI should be 6.0 mm [2,3]. This approach has been reproduced in a great deal of research [12,13,14]. However, some studies suggest that targeting an ILD < 5 mm may be superior to the ILD defined in the CLOSE protocol when ablating with the Thermocool Smarttouch, Thermocool Smarttouch SF [4,5,6,7,8] or a QDot Micro catheter (BW) [9]. This was based on the presumption that typical atrial lesion was 5 mm in diameter [8]. In fact, no randomized studies have been performed to assess this issue. Only Hoffman et al. showed that targeting an ILD of 3–4 mm was superior to an ILD of 5–6 mm, although only with respect to first-pass PVI [5]. It seems that lower ILD values do not necessarily translate into better ablation outcomes. The mean ILD of 4.1 in the CLOSE pilot study resulted in 91% freedom from AF at 12 months [2], whereas the mean ILD of 5.3 mm resulted in an 89% success rate in the VISTAX study [3]. The role of ILD has been also evaluated for other LA procedures. ILD ≤ 6 mm combined with the target AI of 500 was sufficient to achieve a bidirectional block along the anterior LA line without acute reconnection [15]. ILD < 4 mm combined with AI value of 450 resulted in 74% first-pass posterior wall isolation. However, first-pass roof line block was achieved in 80% of cases and the first-pass bottom line block in 83% [16].

Maximum tolerated ILD ≤ 6 mm applied in this study resulted in similar ablation outcomes for both PAF and PsAF populations compared with contemporary studies [2,3,4,5,6,7,8,9,12,13,14]. This suggests that standard ILD values ensure a high level of ablation lesion contiguity. This was confirmed by the fact that global and segmental median ILD did not correlate with AFR in this study. On the other hand, it seems that an optimal maximum ILD at the posterior aspect of RUPV should not exceed 5.5 mm, as suggested by correlation with AFR. This suggests that 5.5–6.0 mm ILD could lead to a virtual or functional interlesion gap within the deployed RF circle at this particular region of RUPV. It is a well-recognized fact that the presence of a single functional gap within ablation encirclement may lead to early PVI failure or further PV reconnection [17]. The underlying mechanism for the detected regional maximum tolerated ILD difference remains unclear. Our personal observations lead to the conclusion that ablation catheter obtains low spatial stability at the posterior aspect of RUPV, probably due to the high local atrial contraction or passive movements. We can speculate that this resulted in the longer ILD than expected. It is also likely that low spatial catheter stability led to inadequate automated lesion annotation and the underestimation of actual ILD. The exact explanation for this phenomenon needs further investigation.

The ablation workflow implemented in this study was different from that defined in the CLOSE protocol [2]. Despite the fact that our approach potentially indicates more intensive ablation, the rate of FPI was relatively low (25–49%). Previously published studies report 87–98% FPI with a standard ablation approach [2,3,4,13]. However, the same workflow resulted in 35% FPI in a study carried out by Hoffman et al. [5].

The results of our study show that even intensive ablation does not guarantee the achievement of FPI. This is unsurprising, as it is impossible to directly visualize both lesion transmurality and contiguity. However, it seems that the key explanation for this result is detailed PVI verification. Although the demonstration of an entrance block into the PV with the Pentaray catheter is sufficient to confirm PVI [1], in our workflow, it was found to be inadequate. In the majority of cases, it did not touch all of the PV segments due to its specific shape. Moreover, information about catheter–tissue contact was missing, meaning no signals or far-field signals were detected. Furthermore, the lack of PV signals does not exclude epicardial capture inside the vein and conduction to LA due to incomplete transmurality along the ablation line. Therefore, we find the lack of capture distal from the ablation line, at each PV segment, assessed with the ablation catheter presenting adequate contact, as a necessary step in PVI assessment.

## 5. Limitations

(a) This was a non-randomized analysis. The next step should be to prospectively confirm the results, especially concerning maximum tolerated ILD at the posterior aspect of RUPV;
(b) AFR could be explained, not only by the discontinuity of ablation lesions, but also by the lack of their transmurality, which was not assessed in this study;
(c) We cannot exclude the fact that steerable sheaths may provide better spatial ablation catheter stability and translate into shorter expected ILD. Furthermore, the adopted ablation strategy in this study (high AI values combined with obligatory impedance drop) might have increased the lesion size compared to other ablation strategies and resulted in the high level of overlapping lesions. Therefore, the outcomes of this study should be interpreted in terms of applied workflow and the ablation catheter employed;
(d) The assessment of ILD was performed postprocedurally. Therefore, ILD calculations included touch-up applications. Intraoperative ILD measurements between all ablation tags greatly prolong the procedure. This step was omitted for that reason. As a consequence, the ILD dataset with regard to achieving or not achieving FPI was missing;
(e) The overall ablation success rate greatly depends on reliable AF recurrence detection. Intermittent rhythm monitoring modalities used in the study were short-term and discontinuous. We cannot rule out that the use of long-term and/or continuous ECG monitoring might have potentially decreased the ablation success rate if it had been applied.

## 6. Conclusions

This study demonstrated that maximum tolerated ILD of 6.0 mm, as defined in the CLOSE protocol, translated into well-accepted ablation results for both paroxysmal and persistent AF patients. However, the study suggests that it may be inadequate at the posterior aspect of RUPV, but adequate elsewhere. Therefore, target ILD < 5.5 mm may be considered at the posterior aspect of RUPV in order to create contiguous lesions and improve results.

## Figures and Tables

**Table 1:** Baseline characteristics of the study cohort and RF point-by-point ablation procedural details among paroxysmal (PAF) and persistent (PsAF) AF subgroups.

	PAF *n* = 130	PsAF *n* = 130	*p*
Uninterrupted AF duration, months	-	24 (12–36)	-
Age, years	58 (53–62)	66 (58–70)	<0.001
Females, *n* (%)	55 (42%)	22 (17%)	<0.001
Hypertension, *n* (%)	82 (63%)	101 (78%)	<0.001
Chronic coronary syndrome, *n* (%)	20 (15%)	31 (24%)	0.02
Heart failure, *n* (%)	3 (2%)	33 (25%)	<0.001
eGFR < 60 mL/min/1.73 m^2^, *n* (%)	3 (2%)	16 (12%)	<0.001
Diabetes, *n* (%)	17 (13%)	29 (22%)	0.03
CHA2DS2–VASc score	2 (1–3)	3 (1–4)	<0.001
Left ventricular ejection fraction, %	60 (55–65)	60 (55–65)	0.8
Left atrial antero-posterior diameter, mm	42 (39–46)	48 (43–55)	<0.001
LCPV, *n*	18 (14%)	21 (16%)	0.4
RMPV, *n*	0	0	-
**Perimeter of encirclement including intravenous carina, mm**			
LUPV	78 (61–94)	103 (93–119)	<0.001
LCPV	81 (63–92)	109 (93–118))	<0.001
LLPV	77 (66–92)	111 (98–123)	<0.001
RUPV	75 (63–91)	109 (96–125)	<0.001
RLPV	72 (59–86)	105 (90–118)	<0.001
**Total number of 6 mm RF tags, *n***			
LUPV	28 (23–34)	34 (29–40)	<0.001
LCPV	32 (29–39)	39 (31–46)	<0.001
LLPV	30 (22–35)	33 (27–38)	<0.001
RUPV	26 (21–33)	35 (29–41)	<0.001
RLPV	26 (22–33)	32 (24–36)	<0.001
**Median interlesion distance, mm**			
LUPV	4.4 (4.2–4.6)	4.5 (4.4–4.7)	0.8
LCPV	3.8 (3.7–4.1)	3.9 (3.8–4.0)	0.9
LLPV	4.4 (4.2–4.5)	4.4 (4.2–4.5)	0.8
RUPV	4.5 (4.3–4.6)	4.6 (4.5–4.7)	0.9
RLPV	4.3 (4.2–4.4)	4.4 (4.3–4.5)	0.8
**Dissociated PV activity following ablation, *n***			
LUPV	18 (14%)	22 (17%)	0.2
LCPV	30 (23%)	35 (27%)	0.6
LLPV	25 (19%)	20 (15%)	0.8
RUPV	66 (51%)	56 (43%)	0.5
RLPV	22 (17%)	25 (19%)	0.6
**First-pass PV isolation, *n***			
LUPV	43 (33%)	39 (30%)	0.5
LCPV	64 (49%)	60 (46%)	0.8
LLPV	32 (25%)	35 (27%)	0.6
RUPV	38 (29%)	32 (25%)	0.4
RLPV	42 (32%)	35 (27%)	0.1

PAF, paroxysmal AF; PsAF, persistent AF; PV, pulmonary vein; LCPV, left common PV; LUPV, left. upper PV; LLPV, left lower PV; RUPV, right upper PV; RLPV, right lower PV, RMPV, right middle PV.

**Table 2:** Differences in number of lesions within each ILD tag group among the AFR and non-AFR group, assessed for paroxysmal AF.

**ILD**	**LCPV: Entire Encirclement**			**LCPV: Anterior Aspect**			**LCPV: Posterior Aspect**					
	**AFR (+)**	**AFR (−)**	***p*** **-Value**	**AFR (+)**	**AFR (−)**	***p*** **-Value**	**AFR (+)**	**AFR (−)**	***p*** **-Value**			
**6.0–5.5**	1 (1–3)	1 (1–2)	0.7	1 (1–1)	1 (0–1)	0.5	1 (1–1)	1 (0–1)	0.7			
**5.5–5.0**	2 (1–4)	1 (1–3)	0.4	1 (1–2)	1 (1–2)	0.7	2 (1–4)	1 (0–1)	0.8			
**5.0–4.5**	3 (2–4)	4 (3–7)	0.7	2 (1–3)	2 (1–4)	0.5	2 (1–4)	2 (2–2)	0.5			
**4.5–4.0**	5 (3–8)	4 (2–8)	0.6	3 (2–6)	2 (2–7)	0.9	3 (1–6)	3 (1–5)	0.6			
**≤4.0**	18 (13–24)	19 (15–28)	0.4	9 (6–18)	9 (7–23)	0.6	10 (8–25)	11 (7–23)	0.8			
**ILD**	**LUPV: Entire Encirclement**			**LUPV: Anterior Aspect**			**LUPV: Posterior Aspect**			**LUPV: Intravenous Carina**		
	**AFR (+)**	**AFR (−)**	***p*** **-Value**	**AFR (+)**	**AFR (−)**	***p*** **-Value**	**AFR (+)**	**AFR (−)**	***p*** **-Value**	**AFR (+)**	**AFR (−)**	***p*** **-Value**
**6.0–5.5**	3 (2–4)	2 (1–3)	0.7	1 (1–2)	1 (1–2)	0.5	2 (1–5)	1 (0–1)	0.9	1 (1–3)	1 (0–1)	0.7
**5.5–5.0**	2 (1–3)	1 (1–3)	0.6	1 (0–1)	0 (0–1)	0.7	1 (1–3)	1 (0–1)	0.7	1 (1–2)	0 (0–1)	0.7
**5.0–4.5**	3 (1–4)	3 (2–4)	0.8	2 (1–4)	2 (1–3)	0.5	1 (1–3)	2 (1–4)	0.5	1 (0–1)	1 (1–2)	0.8
**4.5–4.0**	13 (7–19)	12 (8–16)	0.5	5 (3–11)	7 (4–8)	0.5	6 (2–9)	7 (2–13)	0.8	1 (1–3)	1 (1–4)	0.6
**≤4.0**	8 (5–15)	9 (6–14)	0.5	3 (1–6)	5 (2–9)	0.6	4 (3–5)	4 (3–6)	0.9	1 (0–1)	1 (1–1)	0.7
**ILD**	**LLPV: Entire Encirclement**			**LLPV: Anterior Aspect**			**LLPV: Posterior Aspect**			**LLPV: Intravenous Carina**		
	**AFR (+)**	**AFR (−)**	***p*** **-Value**	**AFR (+)**	**AFR (−)**	***p*** **-Value**	**AFR (+)**	**AFR (−)**	***p*** **-Value**	**AFR (+)**	**AFR (−)**	***p*** **-Value**
**6.0–5.5**	1 (1–3)	1 (0–2)	0.6	1 (0–1)	0 (0–1)	0.7	0 (0–1)	1 (0–2)	0.8	1 (1–4)	1 (1–1)	0.7
**5.5–5.0**	1 (1–1)	2 (1–2)	0.5	1 (0–1)	1 (1–2)	0.7	1 (1–1)	2 (1–3)	0.5	1 (0–1)	0 (0–1)	0.8
**5.0–4.5**	1 (1–3)	1 (0–1)	0.7	0 (0–1)	1 (0–2)	0.8	1 (0–1)	1 (1–2)	0.7	1 (0–1)	1 (0–2)	0.7
**4.5–4.0**	16 (11–20)	14 (11–18)	0.5	8 (3–10)	9 (3–12)	0.7	8 (5–8)	6 (5–7)	0.6	2 (1–3)	2 (1–3)	0.5
**≤4.0**	12 (9–19)	14 (11–17)	0.4	7 (5–9)	8 (6–10)	0.7	6 (4–10)	6 (4–10)	0.6	1 (0–2)	1 (1–2)	0.7
**ILD**	**RUPV: Entire Encirclement**			**RUPV: Anterior Aspect**			**RUPV: Posterior Aspect**			**RUPV: Intravenous Carina**		
	AFR (+)	**AFR (−)**	***p*** **-Value**	**AFR (+)**	**AFR (−)**	***p*** **-Value**	**AFR (+)**	**AFR (−)**	***p*** **-Value**	**AFR (+)**	**AFR (−)**	***p*** **-Value**
**6.0–5.5**	3 (2–7)	1 (0–2)	0.2	0 (0–1)	1 (0–1)	0.8	3 (2–7)	0 (0–1)	<0.001	0 (0–1)	0 (0–1)	0.8
**5.5–5.0**	2 (1–3)	2 (1–4)	0.8	1 (1–2)	1 (0–1)	0.9	0 (0–0)	0 (0–1)	0.8	1 (1–2)	1 (1–1)	0.8
**5.0–4.5**	5 (2–10)	5 (2–9)	0.8	5 (2–8)	6 (3–10)	0.6	0 (0–1)	1 (0–1)	0.7	1 (1–2)	1 (0–2)	0.7
**4.5–4.0**	8 (6–15)	12 (8–14)	0.5	6 (2–9)	7 (2–12)	0.8	3 (1–6)	3 (1–7)	0.8	2 (1–5)	2 (2–5)	0.6
**≤4.0**	6 (3–12)	8 (3–14)	0.5	1 (1–3)	2 (1–5)	0.7	3 (2–7)	6 (3–10)	0.6	2 (1–4)	2 (1–4)	0.9
**ILD**	**RLPV: Entire Encirclement**			**RLPV: Anterior Aspect**			**RLPV: Posterior Aspect**			**RLPV: Intravenous Carina**		
	**AFR (+)**	**AFR (−)**	***p*** **-Value**	**AFR (+)**	**AFR (−)**	***p*** **-Value**	**AFR (+)**	**AFR (−)**	***p*** **-Value**	**AFR (+)**	**AFR (−)**	***p*** **-Value**
**6.0–5.5**	1(1–3)	2 (1–2)	0.7	0 (0–1)	1 (0–1)	0.5	1 (0–1)	0 (0–1)	0.7	1 (1–2)	0 (0–1)	0.7
**5.5–5.0**	0 (0–1)	1 (1–3)	0.6	1 (0–1)	1 (0–1)	0.7	0 (0–1)	1 (1–2)	0.6	0 (0–1)	1 (0–1)	0.5
**5.0–4.5**	1 (1–3)	2 (1–4)	0.6	0 (0–2)	1 (1–2)	0.7	1 (1–1)	1 (1–3)	0.8	1 (0–1)	2 (1–2)	0.9
**4.5–4.0**	14 (9–17)	15 (9–22)	0.8	5 (2–10)	5 (2–10)	0.9	5 (2–9)	5 (3–11)	0.8	3 (1–5)	2 (1–2)	0.7
**≤4.0**	8 (6–11)	7 (6–18)	0.8	5 (2––7)	4 (1–7)	0.6	2 (1–4)	3 (2–7)	0.6	2 (1–3)	2 (1–2)	0.6

ILD, interlesion distance; AFR, AF recurrence; LCPV, left common PV; LUPV, left upper PV; LLPV, left lower PV; RUPV, right upper PV; RLPV, right lower PV.

**Table 3:** Differences in number of lesions within each ILD tag group among the AFR and non-AFR group, assessed for persistent AF.

**ILD**	**LCPV: Entire Encirclement**			**LCPV: Anterior Aspect**			**LCPV: Posterior Aspect**					
	**AFR (+)**	**AFR (−)**	***p*** **-Value**	**AFR (+)**	**AFR (−)**	***p*** **-Value**	**AFR (+)**	**AFR (−)**	***p*** **-Value**			
**6.0–5.5**	2 (2–3)	1 (0–2)	0.4	1 (1–1)	0 (0–1)	0.2	0 (0–1)	0 (0–1)	0.9			
**5.5–5.0**	2 (1–4)	1 (0–3)	0.3	1 (1–1)	1 (0–2)	0.5	1 (0–2)	0 (0–1)	0.3			
**5.0–4.5**	4 (2–6)	4 (3–6)	0.7	2 (1–2)	2 (1–4)	0.5	2 (0–4)	2 (2–2)	0.4			
**4.5–4.0**	2 (1–3)	4 (3–5)	0.2	2 (1–3)	2 (2–3)	0.9	0 (0–0)	1 (1–2)	0.2			
**≤4.0**	27 (22–34)	32 (28–38)	0.3	16 (14–20)	18 (16–23)	0.2	17 (15–21)	21 (17–23)	0.2			
**ILD**	**LUPV: Entire Encirclement**			**LUPV: Anterior Aspect**			**LUPV: Posterior Aspect**			**LUPV: Intravenous Carina**		
	**AFR (+)**	**AFR (−)**	***p*** **-Value**	**AFR (+)**	**AFR (−)**	***p*** **-Value**	**AFR (+)**	**AFR (−)**	***p*** **-Value**	**AFR (+)**	**AFR (−)**	***p*** **-Value**
**6.0–5.5**	2 (1–3)	2 (1–4)	0.5	0 (0–1)	1 (0–2)	0.2	0 (0–1)	0 (0–1)	0.8	1 (1–3)	1 (0–1)	0.6
**5.5–5.0**	2 (1–3)	2 (1–3)	0.5	0 (0–1)	0 (0–2)	0.7	1 (0–1)	1 (0–1)	0.9	0 (0–1)	0 (0–1)	0.8
**5.0–4.5**	3 (1–4)	3 (2–4)	0.6	2 (0–2)	1 (0–2)	0.3	1 (0–1)	1 (0–2)	0.8	1 (0–1)	1 (0–2)	0.8
**4.5–4.0**	15 (12–18)	13 (12–15)	0.3	8 (5–9)	7 (5–8)	0.5	1 (0–2)	1 (0–2)	0.7	5 (4–6)	6 (4–7)	0.5
**≤4.0**	11 (7–21)	12 (8–19)	0.4	4 (3–7)	6 (4–8)	0.2	4 (3–5)	4 (3–6)	0.8	1 (0–1)	1 (0–1)	0.9
**ILD**	**LLPV: Entire Encirclement**			**LLPV: Anterior Aspect**			**LLPV: Posterior Aspect**			**LLPV: Intravenous Carina**		
	**AFR (+)**	**AFR (−)**	***p*** **-Value**	**AFR (+)**	**AFR (−)**	***p*** **-Value**	**AFR (+)**	**AFR (−)**	***p*** **-Value**	**AFR (+)**	**AFR (−)**	***p*** **-Value**
**6.0–5.5**	1 (0–3)	1 (0–2)	0.8	0 (0–1)	0 (0–1)	0.7	0 (0–1)	0 (0–1)	0.8	1 (1–3)	1 (1–1)	0.6
**5.5–5.0**	1 (0–2)	1 (0–2)	0.7	0 (0–1)	0 (0–1)	0.9	0 (0–1)	0 (0–1)	0.8	0 (0–1)	0 (0–1)	0.9
**5.0–4.5**	1 (0–3)	2 (0–4)	0.5	0 (0–1)	1 (0–2)	0.7	1 (0–1)	1 (0–2)	0.7	1 (0–1)	1 (0–2)	0.7
**4.5–4.0**	10 (8–13)	12 (11–14)	0.6	4 (3–7)	5 (3–8)	0.8	6 (5–8)	6 (5–7)	0.6	2 (1–3)	3 (1–3)	0.6
**≤4.0**	16 (13–19)	14 (11–17)	0.5	7 (5–9)	8 (6–10)	0.7	7 (4–8)	7 (5–9)	0.5	2 (1–2)	2 (1–2)	0.6
**ILD**	**RUPV: Entire Encirclement**			**RUPV: Anterior Aspect**			**RUPV: Posterior Aspect**			**RUPV: Intravenous Carina**		
	**AFR (+)**	**AFR (−)**	***p*** **-Value**	**AFR (+)**	**AFR (−)**	***p*** **-Value**	**AFR (+)**	**AFR (−)**	***p*** **-Value**	**AFR (+)**	**AFR (−)**	***p*** **-Value**
**6.0–5.5**	6 (2–7)	2 (0–2)	0.01	1 (0–1)	0 (0–1)	0.4	4 (2–6)	0 (0–1)	<0.001	1 (1–2)	1 (1–2)	0.7
**5.5–5.0**	1 (1–3)	2 (1–3)	0.5	1 (0–2)	1 (0–1)	0.5	0 (0–0)	1 (0–1)	0.7	0 (0–1)	1 (0–1)	0.8
**5.0–4.5**	8 (4–10)	9 (6–12)	0.4	1 (0–3)	1 (0–2)	0.5	0 (0–1)	1 (0–2)	0.7	1 (0–2)	1 (0–2)	0.7
**4.5–4.0**	8 (6–15)	12 (11–14)	0.3	6 (1–3)	7 (1–2)	0.6	1 (0–2)	2 (1–2)	0.6	3 (1–4)	4 (2–6)	0.4
**≤4.0**	12 (6–14)	10 (8–16)	0.5	3 (1–3)	4 (1–5)	0.4	10 (7–12)	8 (6–10)	0.5	2 (1–3)	2 (1–2)	0.2
**ILD**	**RLPV: Entire Encirclement**			**RLPV: Anterior Aspect**			**RLPV: Posterior Aspect**			**RLPV: Intravenous Carina**		
	**AFR (+)**	**AFR (−)**	***p*** **-Value**	**AFR (+)**	**AFR (−)**	***p*** **-Value**	**AFR (+)**	**AFR (−)**	***p*** **-Value**	**AFR (+)**	**AFR (−)**	***p*** **-Value**
**6.0–5.5**	1(1–3)	2 (1–3)	0.6	0 (0–1)	1 (0–1)	0.6	1 (1–1)	1 (0–1)	0.7	1 (1–2)	1 (0–2)	0.7
**5.5–5.0**	1 (0–2)	2 (1–3)	0.5	0 (0–1)	1 (0–1)	0.6	1 (0–1)	1 (0–2)	0.6	0 (0–1)	1 (0–1)	0.4
**5.0–4.5**	2 (1–4)	3 (1–4)	0.6	1 (1–2)	1 (1–2)	0.7	1 (0–1)	2 (1–3)	0.8	1 (0–2)	1 (1–2)	0.6
**4.5–4.0**	15 (11–18)	16 (11–20)	0.7	7 (3–9)	6 (4–9)	0.4	6 (4–9)	6 (3–9)	0.8	3 (1–4)	2 (1–2)	0.7
**≤4.0**	11 (7–12)	10 (6–18)	0.8	5 (2––8)	5 (3–8)	0.6	3 (1–4)	4 (2–5)	0.6	2 (1–4)	2 (1–3)	0.5

See Table 1 for legend details.

**Table 4:** Differences in median ILD among the AFR and non-AFR group, assessed for paroxysmal AF.

	**LCPV: Entire Encirclement**			**LCPV: Anterior Aspect**			**LCPV: Posterior Aspect**					
	**AFR (+)**	**AFR (−)**	***p*** **-Value**	**AFR (+)**	**AFR (−)**	***p*** **-Value**	**AFR (+)**	**AFR (−)**	***p*** **-Value**			
**ILD, mm**	3.8 (3.6–4.0)	3.8 (3.6–3.9)	0.8	3.9 (3.8–4.0)	3.9 (3.7–4.0)	0.7	3.9 (3.7–4.0)	3.8 (3.6–3.9)	0.5			
	**LUPV: Entire Encirclement**			**LUPV: Anterior Aspect**			**LUPV: Posterior Aspect**			**LUPV: Intravenous Carina**		
	**AFR (+)**	**AFR (−)**	***p*** **-Value**	**AFR (+)**	**AFR (−)**	***p*** **-Value**	**AFR (+)**	**AFR (−)**	***p*** **-Value**	**AFR (+)**	**AFR (−)**	***p*** **-Value**
**ILD, mm**	4.5 (4.4–4.7)	4.4 (4.3–4.6)	0.7	4.5 (4.4–4.7)	4.4 (4.3–4.5)	0.6	4.4 (4.4–4.6)	4.5 (4.4–4.7)	0.8	4.5 (4.3–4.5)	4.5 (4.4–4.6)	0.8
	**LLPV: Entire Encirclement**			**LLPV: Anterior Aspect**			**LLPV: Posterior Aspect**			**LLPV: Intravenous Carina**		
	**AFR (+)**	**AFR (−)**	***p*** **-Value**	**AFR (+)**	**AFR (−)**	***p*** **-Value**	**AFR (+)**	**AFR (−)**	***p*** **-Value**	**AFR (+)**	**AFR (−)**	***p*** **-Value**
**ILD, mm**	4.4 (4.1–4.5)	4.4 (4.2–4.6)	0.8	4.5 (4.3–4.6)	4.4 (4.3–4.6)	0.7	4.3 (4.1–4.5)	4.3 (4.2–.4.4)	0.7	4.5 (4.2–4.7)	4.4 (4.2–4.6)	0.6
	**RUPV: Entire Encirclement**			**RUPV: Anterior Aspect**			**RUPV: Posterior Aspect**			**RUPV: Intravenous Carina**		
	**AFR (+)**	**AFR (−)**	***p*** **-Value**	**AFR (+)**	**AFR (−)**	***p*** **-Value**	**AFR (+)**	**AFR (−)**	***p*** **-Value**	**AFR (+)**	**AFR (−)**	***p*** **-Value**
**ILD, mm**	4.5 (4.3–4.7)	4.5 (4.4–4.7)	0.7	4.5 (4.3–4.7)	4.6 (4.4–4.6)	0.7	4.5 (4.3–4.6)	4.6 (4.3–4.5)	0.6	4.5 (4.3–4.6)	4.4 (4.2–4.6)	0.5
	**RLPV: Entire Encirclement**			**RLPV: Anterior Aspect**			**RLPV: Posterior Aspect**			**RLPV: Intravenous Carina**		
	**AFR (+)**	**AFR (−)**	***p*** **-Value**	**AFR (+)**	**AFR (−)**	***p*** **-Value**	**AFR (+)**	**AFR (−)**	***p*** **-Value**	**AFR (+)**	**AFR (−)**	***p*** **-Value**
**ILD, mm**	4.3 (4.1–4.5)	4.3 (4.1–4.5)	0.8	4.4 (4.2–4.7)	4.4 (4.2–4.5)	0.7	4.3 (4.1–4.6)	4.4 (4.2–4.6)	0.7	4.4 (4.3–.4.5)	4.4 (4.3–4.6)	0.7

See Table 1 for legend details.

**Table 5:** Differences in median ILD among the AFR and non-AFR groups, assessed for persistent AF.

	**LCPV: Entire Encirclement**			**LCPV: Anterior Aspect**			**LCPV: Posterior Aspect**					
	**AFR (+)**	**AFR (−)**	***p*** **-Value**	**AFR (+)**	**AFR (−)**	***p*** **-Value**	**AFR (+)**	**AFR (−)**	***p*** **-Value**			
**ILD, mm**	3.9 (3.7–4.0)	3.8 (3.6–4.0)	0.8	3.9 (3.8–4.0)	3.9 (3.7–4.0)	0.7	3.9 (3.7–4.0)	3.8 (3.6–3.9)	0.5			
	**LUPV: Entire Encirclement**			**LUPV: Anterior Aspect**			**LUPV: Posterior Aspect**			**LUPV: Intravenous Carina**		
	**AFR (+)**	**AFR (−)**	***p*** **-Value**	**AFR (+)**	**AFR (−)**	***p*** **-Value**	**AFR (+)**	**AFR (−)**	***p*** **-Value**	**AFR (+)**	**AFR (−)**	***p*** **-Value**
**ILD, mm**	4.5 (4.4–4.7)	4.5 (4.3–4.6)	0.6	4.5 (4.4–4.6)	4.4 (4.3–4.5)	0.7	4.5 (4.4–4.6)	4.5 (4.4–4.7)	0.8	4.4 (4.3–4.5)	4.5 (4.4–4.6)	0.7
	**LLPV: Entire Encirclement**			**LLPV: Anterior Aspect**			**LLPV: Posterior Aspect**			**LLPV: Intravenous Carina**		
	**AFR (+)**	**AFR (−)**	***p*** **-Value**	**AFR (+)**	**AFR (−)**	***p*** **-Value**	**AFR (+)**	**AFR (−)**	***p*** **-Value**	**AFR (+)**	**AFR (−)**	***p*** **-Value**
**ILD, mm**	4.3 (4.2–4.5)	4.4 (4.3–4.5)	0.5	4.4 (4.3–4.5)	4.4 (4.3–4.5)	0.7	4.4 (4.3–4.5)	4.3 (4.2–4.4)	0.3	4.5 (4.4–4.7)	4.4 (4.3–4.5)	0.4
	**RUPV: Entire Encirclement**			**RUPV: Anterior Aspect**			**RUPV: Posterior Aspect**			**RUPV: Intravenous Carina**		
	**AFR (+)**	**AFR (−)**	***p*** **-Value**	**AFR (+)**	**AFR (−)**	***p*** **-Value**	**AFR (+)**	**AFR (−)**	***p*** **-Value**	**AFR (+)**	**AFR (−)**	***p*** **-Value**
**ILD, mm**	4.6 (4.4–4.7)	4.5 (4.4–4.6)	0.6	4.6 (4.4–4.7)	4.5 (4.4–4.6)	0.7	4.6 (4.5–4.7)	4.4 (4.3–4.5)	0.4	4.5 (4.3–4.6)	4.5 (4.4–4.6)	0.6
	**RLPV: Entire Encirclement**			**RLPV: Anterior Aspect**			**RLPV: Posterior Aspect**			**RLPV: Intravenous Carina**		
	**AFR (+)**	**AFR (−)**	***p*** **-Value**	**AFR (+)**	**AFR (−)**	***p*** **-Value**	**AFR (+)**	**AFR (−)**	***p*** **-Value**	**AFR (+)**	**AFR (−)**	***p*** **-Value**
**ILD, mm**	4.4 (4.3–4.6)	4.3 (4.2–4.5)	0.6	4.3 (4.2–4.6)	4.4 (4.2–4.5)	0.5	4.4 (4.2–4.6)	4.4 (4.2–4.5)	0.7	4.4 (4.3–.4.5)	4.3 (4.3–4.5)	0.6

See Table 1 for legend details.

## Data Availability

The data presented in this study are available on request. The data are not publicly available due to ethical restrictions.

## Supplementary Materials

The following supporting information can be downloaded at: https://www.mdpi.com/article/10.3390/jcm12155056/s1, Figure S1: A receiver operating characteristic (ROC) curve analysis was used to evaluate the predictive value of the presence of 5.5–6.0 mm tags located on the posterior aspect of Right Upper Pulmonary Vein (RUPV) for a postablation AF recurrence among paroxysmal AF subgroup; Figure S2: A receiver operating characteristic (ROC) curve analysis was used to evaluate the predictive value of the presence of 5.5–6.0 mm tags located on the posterior aspect of Right Upper Pulmonary Vein (RUPV) for a postablation AF recurrence among persistent AF subgroup.

## References

[1] Calkins H., Hindricks G., Cappato R., Kim Y.-H., Saad E.B., Aguinaga L., Akar J.G., Badhwar V., Brugada J., Camm J. (2017). 2017 HRS/EHRA/ECAS/APHRS/SOLAECE expert consensus statement on catheter and surgical ablation of atrial fibrillation. Heart Rhythm..

[2] Taghji P., El Haddad M., Phlips T., Wolf M., Knecht S., Vandekerckhove Y., Tavernier R., Nakagawa H., Duytschaever M. (2018). Evaluation of a Strategy Aiming to Enclose the Pulmonary Veins With Contiguous and Optimized Radiofrequency Lesions in Paroxysmal Atrial Fibrillation: A Pilot Study. JACC Clin. Electrophysiol..

[3] Duytschaever M., Vijgen J., De Potter T., Scherr D., Van Herendael H., Knecht S., Kobza R., Berte B., Sandgaard N., Albenque J.-P. (2020). Standardized pulmonary vein isolation workflow to enclose veins with contiguous lesions: The multicentre VISTAX trial. Europace.

[4] Hussein A., Das M., Chaturvedi V., Asfour I.K., Daryanani N., Morgan M., Ronayne C., Shaw M., Snowdon R., Gupta D. (2017). Prospective use of Ablation Index targets improves clinical outcomes following ablation for atrial fibrillation. J. Cardiovasc. Electrophysiol..

[5] Hoffmann P., Ramirez I.D., Baldenhofer G., Stangl K., Mont L., Althoff T.F. (2020). Randomized study defining the optimum target interlesion distance in ablation index-guided atrial fibrillation ablation. Europace.

[6] Theis C., Huber C., Kaiser B., Kaesemann P., Hui F., Pirozzolo G., Bekeredjian R. (2021). Improved durable pulmonary vein isolation with shorter procedure times and lower energy levels using RF ablation with ablation index and a stringent lesion contiguity. Indian Pacing Electrophysiol. J..

[7] Yazaki K., Ejima K., Kataoka S., Kanai M., Higuchi S., Sh S.H., Shoda M., Hagiwara N. (2021). Regional differences in the predictors of acute electrical reconnection following high-power pulmonary vein isolation for paroxysmal atrial fibrillation. J. Arrhythmia.

[8] Jankelson L., Dai M., Aizer A., Bernstein S., Park D.S., Holmes D., Chinitz L.A., Barbhaiya C. (2021). Lesion Sequence and Catheter Spatial Stability Affect Lesion Quality Markers in Atrial Fibrillation Ablation. JACC Clin. Electrophysiol..

[9] Mitrzak K., Peller M., Krzowski B., Maciejewski C., Balsam P., Marchel M., Grabowski M., Lodziński P. (2023). Safety and effectiveness of very-high-power, short-duration ablation in patients with atrial fibrillation: Preliminary results. Cardiol. J..

[10] Kiedrowicz R.M., Wielusinski M., Wojtarowicz A., Kazmierczak J. (2022). Predictors of the voltage derived left atrial fibrosis in patients with long-standing persistent atrial fibrillation. Cardiol. J..

[11] Takigawa M., Yamada T., Yoshida Y., Ishikawa K., Aoyama Y., Yamamoto T., Inoue N., Tatematsu Y., Nanasato M., Kato K. (2012). The incidence and clinical significance of non-isolation of the pulmonary vein carina after encircling ipsilateral pulmonary veins isolation for paroxysmal atrial fibrillation: A pitfall of the double-Lasso technique. Europace.

[12] Dhillon G., Ahsan S., Honarbakhsh S., Lim W., Baca M., Graham A., Srinivasan N., Sawhney V., Sporton S., Schilling R.J. (2019). A multicentered evaluation of ablation at higher power guided by ablation index: Establishing ablation targets for pulmonary vein isolation. J. Cardiovasc. Electrophysiol..

[13] Berte B., Hilfiker G., Moccetti F., Schefer T., Weberndörfer V., Cuculi F., Toggweiler S., Ruschitzka F., Kobza R. (2019). Pulmonary vein isolation using ablation index vs. CLOSE protocol with a surround flow ablation catheter. Europace.

[14] Kiliszek M., Krzyżanowski K., Wierzbowski R., Winkler A., Smalc-Stasiak M. (2020). The value of the ablation index in patients undergoing ablation for atrial fibrillation. Kardiologia Polska.

[15] Santoro F., Metzner A., Brunetti N.D., Heeger C.-H., Mathew S., Reissmann B., Lemeš C., Maurer T., Fink T., Rottner L. (2019). Left atrial anterior line ablation using ablation index and inter-lesion distance measurement. Clin. Res. Cardiol..

[16] Makihara Y., Miyazaki S., Harama T., Obunai K., Watanabe H., Tada H. (2022). Ablation Index Guided Left Atrial Posterior Wall Isolation. Int. Heart J..

[17] Jiang R.-H., Jiang C.-Y. (2016). Pulmonary Vein Reconnection in Patients With and Without Atrial Fibrillation Recurrence After Ablation. JACC Clin. Electrophysiol..

