# Supplementary material for: The Impact of Regional Maximum Tolerated Interlesion Distance on the Long-Term Ablation Outcomes in Ablation Index Guided Pulmonary Vein Isolation for Atrial Fibrillation
Source: J Clin Med. 2023 Aug 1;12(15):5056. doi: 10.3390/jcm12155056 (PMC10420066; doi:10.3390/jcm12155056)

Journal of
Clinical Medicine

MDPI

Figure S1. A receiver operating characteristic (ROC) curve analysis was used to evaluate the predictive value of the presence of 5.5–6.0 mm tags located on the posterior aspect of Right Upper Pulmonary Vein (RUPV) for a postablation AF recurrence among paroxysmal AF subgroup.

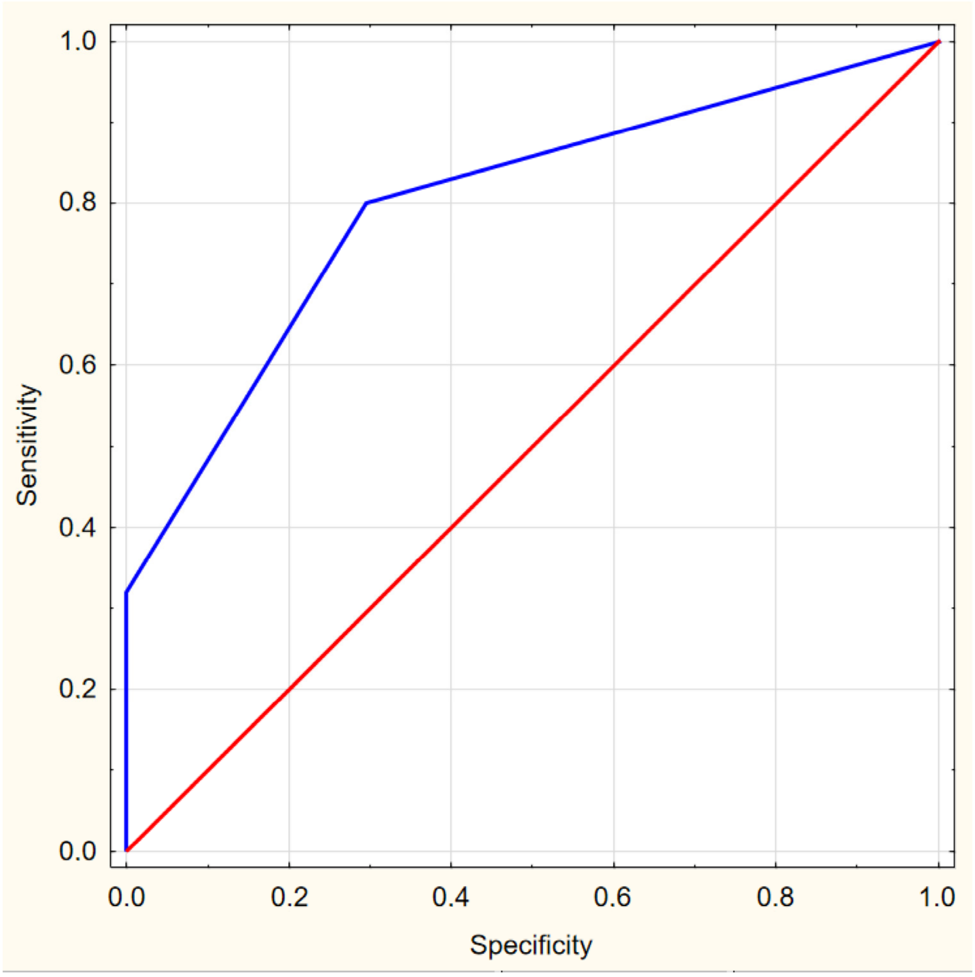

Figure S2. A receiver operating characteristic (ROC) curve analysis was used to evaluate the predictive value of the presence of 5.5–6.0 mm tags located on the posterior aspect of Right Upper Pulmonary Vein (RUPV) for a postablation AF recurrence among persistent AF subgroup.

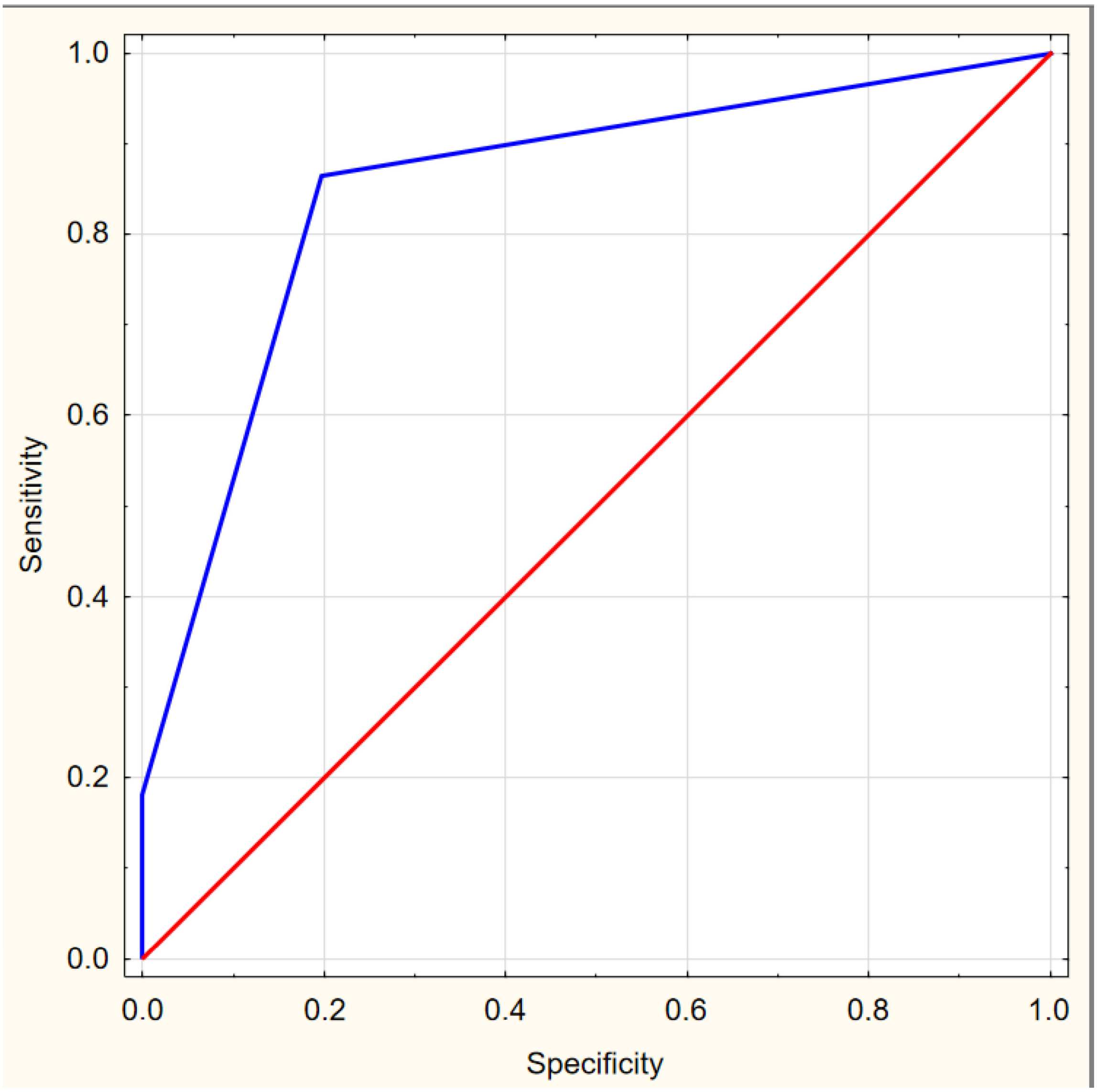

Supplement: Supplementary file 1 [file jcm-12-05056-s001.zip › jcm-2394886-supplementary.pdf]
